# Potential therapeutic strategy for non-Hodgkin lymphoma by anti-CD20scFvFc/CD28/CD3zeta gene tranfected T cells

**DOI:** 10.1186/1756-9966-29-121

**Published:** 2010-09-03

**Authors:** Yihu Zheng, Kang Yu, Jimei Du, Lei Jiang, Shenghui Zhang, Yixiang Han, Panpan Yu, Yingxia Tan

**Affiliations:** 1 Institute of Medical Research, The First Affiliated Hospital of Wenzhou Medical College, Wenzhou 325000, China; 2 Department of Gerenal Surgery, The First Affiliated Hospital of Wenzhou Medical College, Wenzhou 325000, China; 3 Institute of Hematology and Immunology, The First Affiliated Hospital of Wenzhou Medical College, Wenzhou 325000, China; 4 Department of Hematology, The First Affiliated Hospital of Wenzhou Medical College, Wenzhou 325000, China

## Abstract

**Background:**

Anti-CD20 monoclonal antibody treatment has not only increased survival and cure rates in many non-Hodgkin lymphomas, but also has prompted an explosion in the development of novel antibodies and biologically active substances with specific cellular targets in the field of malignancies treatment. Since the robust immune responses are elicited by the gene-modified T cells, gene based T cell therapy may also provide a powerful tool for cancer immunotherapy.

**Methods:**

In this study, we developed a vector construction encoding a chimeric T cell receptor that recognizes the CD20 antigen and delivers co-stimulatory signals to achieve T cell activation. One non-Hodgkin lymphoma cell line Raji cells co-cultured with peripheral blood-derived T cells were stably transfected with anti-CD20scFvFc/CD28/CD3zeta gene or anti-CD20scFvFc gene. T cells expressing anti-CD20scFvFc/CD28/CD3zeta or anti-CD20scFvFc gene co-cultured with CD20 positive Raji cells for different times. Cell lysis assay was carried by [^3^H]TdR release assay. The expressions of Fas, Bcl-2 and Caspase-3 of Raji cells were detected by flow cytometric. The secretion of IFN-gamma and IL-2 in co-culture medium was tested by ELISA assay. Activity of AP-1 was analyzed by EMSA.

**Results:**

Following efficient transduction of peripheral blood-derived T cells with anti-CD20scFvFc/CD28/CD3zeta gene, an obvious cell lysis of Raji cells was observed in co-culture. T cells transduced anti-CD20scFvFc/CD28/CD3zeta gene had superior secretion of IFN-gamma and IL-2 compared to T cells transduced anti-CD20scFvFc gene. Also it led to a much stronger Fas-induced apoptosis signaling transduction in target cancer cells.

**Conclusion:**

So adoptively T cells transduced anti-CD20scFvFc/CD28/CD3zeta gene mediates enhanced anti-tumor activities against CD20 positive tumor cells, suggesting a potential of gene-based immunotherapy for non-Hodgkin lymphoma.

## Background

Non-Hodgkin's lymphoma, known as one of hematologic malignancies, is aggressive tumor with a poor prognosis. Although the clinical outcome of the patients has improved dramatically with combination chemotherapy (CHOP and other standard protocols) and anti-CD20 monoclonal antibody therapy, non-Hodgkin's lymphoma has been proved to be refractory or relapse, and is ultimately failure to standard treatments [1]. Therefore, various strategies have been proposed to treat Non-Hodgkin's lymphoma. Adoptive immunotherapy with genetically modified T cells expressing cTCRs targeting lymphoma-associated antigens appears to be a promising candidate. These receptors all consist of an Ag-binding domain, which is connected to a trans-membrane domain, and fused to an intracellular signaling domain. The extracellular Ag-binding domain most usually consists of the scFv region of an antibody against the target antigen. The common used intracellular signaling region with the most potential is the CD3ζ chain. It had been previously shown to be sufficient for mediating T cell activation signals [2]. But it has recently become increasingly clear that successful adoptive T cell therapy requires co-stimulation: without adequate co-stimulatory signals, resting peripheral T cells can not become activated through an intracellular ζ chain alone [3]. However, as a means of immune escape, tumors do not express or down-regulate co-stimulatory ligands [4]. Subsequent studies found enforced expression of a CD28 signaling domain linked to a scFv Ag-binding region successfully provided co-stimulation. It allowed T cells to become activated, escape pro-apoptotic conditions, and preferentially expand in culture compared to unmodified cells [5].

In this article, we describe a vector encoding a chimeric T-cell receptor binding the antigen CD20. The vector construction has been described in detail by Yu et al [6]. The advantage of this particular construction is that it contains a co-stimulatory signaling motif from the CD28 co-receptor. It has previously been demonstrated to enhance T cell activation [5]. We have recently described activity of gene-modified T cells expressing a chimeric receptor targeting CD20 against hematological tumors [6]. But the correlative mechanism of T cells grafted with this recombinant gene to lyse target tumor cells has not been elucidated. Our experiments are designed to provide new clew for this recombinant gene modified T cells against CD20 positive B-cell non-Hodgkin lymphoma.

## Materials and methods

### Culture medium

RPMI 1640 Medium containing 2 mmol/L of L-glutamine, 25 mmol/L of Hepes (GIBCO, Groud Island, NY), and 10% FBS (Bio international New Zealand) was used for Raji and Peripheral Blood Mononuclear Cell (PBMC) culture.

### Cell line

Fresh human peripheral mononuclear cells obtained from normal healthy donors. Burkitt lymphoma cell line Raji obtained from ATCC. Cells were cultured in a humidified atmosphere containing 5% CO2 at 37°C.

### Experimental protocol

The subjects were assigned into three groups: blank group (untransfected T cells co-cultured with Raji cells), control group (T cells transduced with anti-CD20scFvFc receptor co-cultured with Raji cells), and experimental group (T cells transduced with anti-CD20scFvFc/CD28/CD3ζ receptor co-cultured with Raji cells). In each group, 2 × 10^6 ^T cells were co-cultured with 2 × 10^5 ^Raji cells at 37°C for indicated time in 6-well plates.

### Plasmid DNA

pLNCX vector containing anti-CD20 scFv was previously provided by Dr. Daming Shan (University of Washington, USA). pBULLET vector containing anticarcinoembryonic antigen (anti-CEA) scFv/CD28/CD3ζ was kindly provided by Dr. Hinrich Abken (Laboratory of Tumor Genetic, Department of Internal Medicine, University of Cologne, Germany). The assembly and confirmation of the anti-CD20scFvFc/CD28/CD3ζ receptor has been previously described. The recombinant plasmids were amplified in Escherichia coli DH5α and linearized by for 4 hours at 37°C incubation with 150 units of ECOR1 (Fermentas USA) for each 100 μg plasmid DNA. The recombinant plasmids were purified by PCR Purification Kits (Qiagen, Germany) after incubated at 65°C for 15 min. The plasmids were dissolved in TE buffer at a concentration of 1 μg/μl and then stored at -20°C until used for electroporation.

### Generation of Recombinant Gene Modified T Cells

Heparinized peripheral blood from normal donors was diluted 1:2 in PBS. PBMCs were isolated by density centrifugation and cultured in RPMI 1640 Medium containing 10% FBS. The Medium was also supplemented with 1 μg/ml PHA-L (Roche, USA), 50 U/ml rhIL-2 (Sigma, USA), and 30 ng/ml OKT3 (Wuhan Institute of Biological Products, China). The recombinant human IL-2 (Sigma, USA) was added to OKT3-activated PTLs after 72 hours initial culture. The aspiration of the culture medium (contain IL-2 and OKT3) was followed every 3 days after 10 days sustainable culture. Thus, 1 × 10^6 ^cells were collected and Lymphocyte subsets assay was analyzed by flow cytometry by using Simultest Imk-Lymphocyte Kit (BD, USA). When the rate of CD3 positive cells was above 90% among PBMCs and the amount of cells numbers exceeded 5 × 10^7^, plasmid transduction of T cells was followed. A mixture of 100 μg plasmid DNA and 10 mg/ml salmon sperm DNA (Invitrogen USA) was made. Then, 5 × 10^7 ^PBMCs were added into RPMI 1640 Medium with the mixture. Cells suspension was aliquoted into 0.4 ml electroporation cuvettes. The plasmid was introduced into activated T lymphocytes by electroporation by using a Multiporator (Bio-Rad Gene Pulser Xcell USA) set at 300 V, 2 ms. Cells were incubated at room temperature for 5 min, resuspended in culture medium (contain 10% FBS, IL-2 and OKT3), and then incubated in an incubator at 37°C in 5% CO_2_. G418 (cALBio-chem USA) at an active concentration of 800 μg/ml was added to culture medium after electroporation for 48 hours. PBMCs were selected by G418 for 7 days prior to cloning. G418-resistant PBMCs were successfully transfected with recombinant gene.

### Western blot analysis

Whole cells lysates of untransfected and transduced with two types of recombinant gene were generated by lysis of 2 × 10^7 ^washed cells in 1 mL of Protein Extraction Reagent (Thermo Scientific USA). Protein electrophoresis, transferring to membrane and blotting were carried out according to standard protocol.

### Microscopy

Microscopic analysis was performed to study morphological alteration of Raji cells. Therefore, experimental and blank groups incubated at 37°C for indicated time in 6-well assay plate were investigated by using microscope (Leica).

### Cell lysis assay

DNA fragmentation induced by gene modified T cells in Raji cells was employed by [^3^H]TdR release assay. 2 × 10^6 ^Raji cells were preincubated with 20 μci [^3^H]TdR (GE healthcare) at 37°C for 4 hours. Each 2 × 10^5 ^Raji cells were co-cultured with 2 × 10^6 ^gene modified or untransfected T cells at 37°C in 6-well plates. 100 μL cell-free supernatants were harvested and mixed with 1 ml scintillation liquid after incubation for indicated time at 37°C. Radioactivity was detected by scintillation counting (Beckman). The percentage of specific lysis was calculated as 100 × [(experimental release)-(spontaneous release)/(maximum release)-(spontaneous release)]. Spontaneous release of [^3^H]TdR by target cells was evaluated in wells containing medium alone. Maximum release value was obtained from target cells incubated with 2% SDS.

### Flow cytometric analysis to determine expression of Fas, Bcl-2 and Caspase-3

Cells from three groups were fixed and permeabilized by Cytofix/Cytoperm reagent (Becton Dickinson PharMingen) after harvested from 6-well assay plates. Then they were indicated by Cy5-conjugated CD20 antibody and a panel of antibodies including PE-conjugated Fas antibody, FITC-conjugated Bcl-2 antibody, and PE-conjugated Caspase-3 antibody for analysis of cell immunophenotypes. Cells were washed twice, resuspended in 300 μl PBS containing 3% paraformaldehyde, and analyzed by using a FACSCalibur (Becton Dickinson) after incubation for 25 min at 37°C.

### Analysis of cytokine production

Cells in three groups were cultured in 6-well assay plates for 24 hours. Thus, cell supernatants were collected, and ELISA assay for IFN-gamma and IL-2 was carried out by using the R&D Systems kit.

### Electrophoretic Mobility Shift Assay (EMSA)

Cells were harvested and washed twice with PBS before staining with Cy5-labeled anti-CD3 antibody and further separated by a FACSCalibur (Becton Dickinson). The nuclear fractionation of T cells was carried out according to the manufacturer's instructions by using the NE-PER Nuclear and Cytoplasmic Extraction Reagents (Pierce Biotechnology). AP-1 DNA binding was assayed using 5'-CGCTTGATGAGTCAGCCGGAA-3' oligonucleotide as a probe. The double stranded, AP-1 oligonucleotide was labeled with biotin. Binding reactions were carried out for 20 min at room temperature in the presence of 50 ng/μl poly(dI-dC), 0.05% Nonidet P-40, 5 mmol/L MgCl_2_, 10 mmol/L EDTA, and 2.5% glycerol in 1 × binding buffer (LightShift™ chemiluminescent EMSA kit, Pierce) using 2 μl of biotin, end labelled target DNA and 5 μg of nuclear extract. Unlabelled target DNA was added to 20 μl of binding reaction where indicated as a negative control. Assays were loaded onto native 6% polyacrylamide gels pre-electrophoresed for 30 minutes in 0.5 × Tris borate/EDTA and electrophoresed at 100 V for 50 minutes. The DNA is then transferred to a positive nylon membrane, UV-crosslinked, probed with horseradish peroxidase conjugated streptavidin (LightShift™ chemiluminescent EMSA kit) according to the manufacturer's instructions.

### Statistical analysis

The results of each series of experiments (performed in triplicates) were expressed as the mean values ± standard deviation of the mean (SD). Statistical significance of differences between groups was analyzed by using ANOVA analysis. *P *< 0.05 was considered statistically significant.

## Results

### Assembly of anti-CD20 scFvFc/CD28/CD3ζ

**Figure 1:**
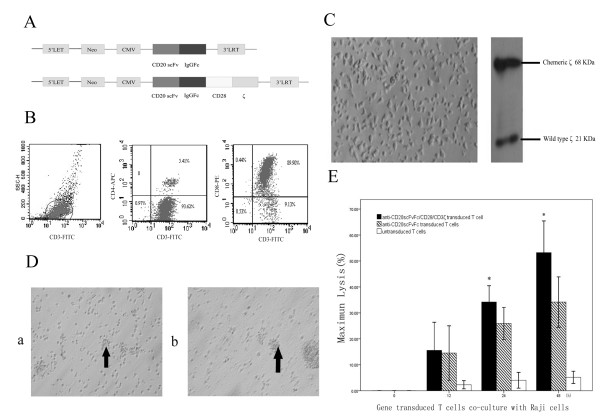
**A: Schematic diagram of the anti-CD20scFvFc-pLNCX and anti-CD20scFvFc/CD28/CD3ζ pLNCX, LTR: long term repeat, Neo: neomycin, CMV: cytomegalovirus**. **B**: The CD3, CD4 and CD8 antigens on surface of PBMCs, which incubated for 10 days after stimulation by PHA-L, OKT3 and IL-2 were analyzed by flow cytometry. A life gate was set around CD3 positive cells; only those cells expressing this membrane protein were included, and 20,000 events were analyzed. **C**: PBMCs grafted with anti-CD20scFvFc/CD28/CD3ζ after selected by G418 for 7 days and analysis of PBMCs grafted with anti-CD20scFvFc/CD28/CD3ζ by Western blot. **D-a:**PBMCs grafted with anti-CD20scFvFc/CD28/CD3ζ co-culture with Raji cells for 12 hours. **D-b**: PBMCs grafted with anti-CD20scFvFc/CD28/CD3ζ co-culture with Raji cells for 24 hours. **E**: Cell lysis evaluated by [3H]TdR release assay. (In experimental group, *represents *p *< 0.05 compared to control group at the same time point).

The whole DNA fragment coding for anti-CD20scFvFc/CD28/CD3ζ was shown in Fig. 1A. It was confirmed by restriction digestion mapping and DNA sequencing.

### Expression of anti-CD20scFvFc/CD28/CD3ζ in PBMCs

T Lymphocyte Subsets of PBMCs was analyzed by flow cytometry. As showed in Fig. 1B, the CD3 positive cell population of PBMCs was above 90% and the CD8 positive CTL cells accounted for the majority of PBMCs population. Cell lysates from transduced peripheral blood T lymphocytes were probed with an anti-CD3ζ mAb to detect the endogenous CD3ζ and the recombinant CD3ζ in transduced PBMCs. As shown in Fig. 1C, a 21 KDa band corresponding to wild-type CD3ζ and a 68 KDa band consistent with anti-CD20scFvFc/CD28/CD3ζ were present in cell lysates of transduced peripheral blood T lymphocytes after 7 days culture.

### Morphology

The Raji cells adhered to T cells, but kept integrity of cell morphology after 2 hours co-culture with anti-CD20scFvFc/CD28/CD3ζ transduced T cells. Raji cells in experimental group presented prominent cell swelling, intramural thickening of granule and cell lysis after 12 hours co-culture (Fig. 1D-a). Raji cells in experimental group showed vast cell death associated with cell split after 24 hours co-culture (Fig. 1D-b). During the whole process, the modified T cells kept in a good integrity of cell morphology.

### Target cell lysis by T cells

The specific killing of CD20-positive Raji cells by T cells transduced anti-CD20scFvFc/CD28/CD3ζ or anti-CD20scFvFc recombinant gene was showed in cytotoxicity assays. But T cells transduced anti-CD20scFvFc/CD28/CD3ζ gene had superior ability to lyse the CD20-positive tumor cells compared to T cells transduced anti-CD20scFvFc gene. There was slight lysis of Raji cells co-cultured with untransduced T cells (Fig. 1E).

### Flow cytometric analysis to determine expression of Fas, Bcl-2 and Caspase-3

**Figure 2:**
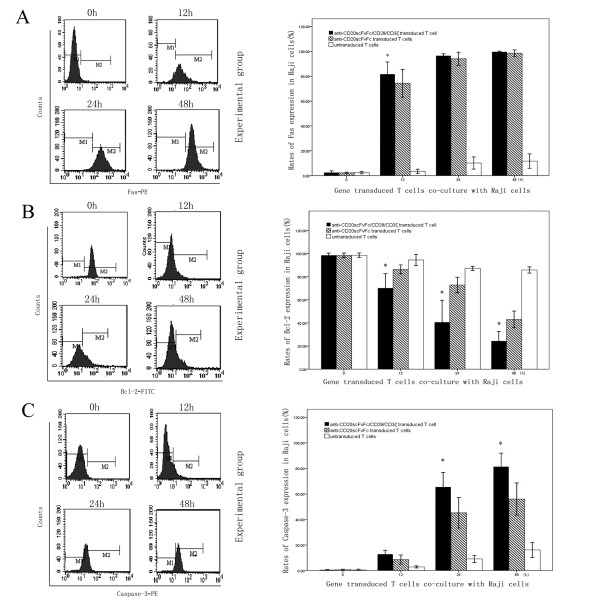
**The co-cultured PBMCs and Raji cells were separated by CD20 expressing**. The CD20 antigens on surface of Raji cells were analyzed by flow cytometry. A life gate was set around CD20 positive cells; only those cells expressing this membrane protein were included, and 20,000 events were analyzed. **A**: The expression of Fas in Raji cells co-cultured with anti-CD20scFvFc/CD28/CD3ζ, anti-CD20scFvFc transduced T cells or untransduced T cells were analyzed by flow cytometry. **B**: The expression of Bcl-2 in Raji cells co-cultured with anti-CD20scFvFc/CD28/CD3ζ, anti-CD20scFvFc transduced T cells or untransduced T cells were analyzed by flow cytometry. **C**: The expression of Caspase-3 in Raji cells co-cultured with anti-CD20scFvFc/CD28/CD3ζ, anti-CD20scFvFc transduced T cells or untransduced T cells were analyzed by flow cytometry. (In experimental group, *represents *p *< 0.05 compared to control group at the same time point).

Although Fas initially had a low basal expression in Raji cells, its expression sharply ascended in experimental and control group after 12 hours co-culture with gene modified T cells. Its expression had a statistically significant difference between experimental and control group at 12-hour time point. After that, the difference became undetectable due to the restriction of the rates of positive expression analyzed by flow cytometric (Fig. 2A).

Raji cells originally had a high basal expression of Bcl-2 response to the positive expression rates above 95%. An obvious downward trend of Bcl-2 expression of Raji cells was observed in experimental and control group compared to blank group. It was noteworthy that Bcl-2 expression of Raji cells in experimental group had an aggressively decline from 12 to 48 hours. During this process, the experimental group showed obviously significant difference compared to the counterparts in control and blank group (*P *< 0.05) (Fig. 2B).

It appeared to be a marked increase in Caspase-3 expression of Raji cells in experimental and control group compared to blank group. Raji cells in experimental group led to a significantly greater proportion of Caspase-3 expression compared to control group and blank group after 12 hours co-culture (Fig. 2C).

### Secretion of IFN-gamma and IL-2

**Figure 3:**
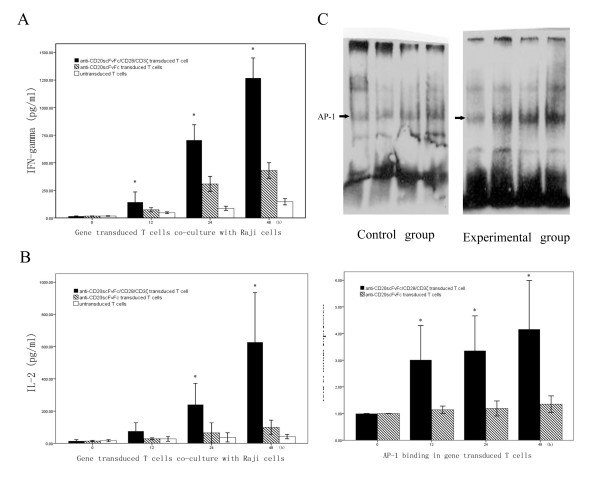
**A: Raji cells were co-cultured with anti-CD20scFvFc/CD28/CD3ζ, anti-CD20scFvFc transduced T cells or untransduced T cells**. Supernatants from these cultures were tested by ELISA for IFN-gama. **B**: Supernatants from these cultures were tested by ELISA for IL-2. C: AP-1 DNA binding were measured by EMSA. (In experimental group, *represents *p *< 0.05 compared to control group at the same time point).

T cells co-cultured with Raji cells could induce a sustaining secretion of IFN-gamma in a time-dependent manner. Comparing to control and blank group, IFN-gamma secreted in experimental group had an express go up at 12-hour time point and was obvious superior in subsequent time points (Fig. 3A).

As the time go by, the secretion of IL-2 in supernatant of experimental group had an obvious increase trend. It had obvious superior statistically significant differences compared to other two groups from initial co-culture (Fig. 3B).

### AP-1 binding in gene modified T cells

Due to it has been demonstrated that there is a strong cooperativity between transcription factors that bind to the IL-2 promoter, in particular, activating protein 1 (AP-1) in regulating IL-2 transcription. To determine if gene modified T cells increase IL-2 secretion levels by altering the DNA binding activity of the transcription factor, AP-1, EMSA analysis was performed. Our results demonstrated that gene modified T cells altered the DNA binding activity of AP-1. AP-1 binding in gene modified T cells of experimental group had distinctly superior compared to control group (Fig. 3C).

## Discussion

The anti-CD20 monoclonal antibody has demonstrated its efficacy in non-Hodgkin's lymphoma treatment. However, despite the success of Rituximab treatment, resistance resulting to non-response to treatment or early relapse of the original disease occurs in around 50% of the patients [7]. Although the precise mechanism of resistance to Rituximab is not fully understood, it is suggested that the patient-specific microenvironment of the lymphoma is related to cancer resistance. The significance of the microenvironment in Rituximab-induced cell death is indirectly observed by differential responses to Rituximab therapy in different subtypes of CD20-positive lymphomas (which have unique microenvironments) [7]. Malignant tumor cells can receive additional survival signals in some unique microenvironments, as some lymph node compartments (germinal centres) [3,8]. Moreover, the myeloid-lineage cells infiltrating some of these lymphomas may provide trophic stimuli to the malignant cells [9]. Exposure to these pro-survival signals makes these cells less sensitive to the anti-CD20 antibody. Accordingly, attempts have been made to improve the therapeutic efficacy and overcome some resistance. For example, combination therapy is a method to overcome some resistance to regular chemotherapy in some patients who over-express Bcl-2 [10]. Meanwhile, other methods are made to improve ADCC by immunostimulatory molecules, such as some cytokines, or enhancing CDC by down-regulation of complement regulatory proteins [7]. Since T cells can transfer to lymph nodes, lyse multiple targets, proliferate in response to antigenic stimulation, and persist in the tumor-bearing host for prolonged periods of time, the modified T cells expressing chimeric T cell receptors targeting lymphoma-associated antigen appear to be a promising alternative [11,12]. Also recent innovations including enhanced co-stimulation, exogenous cytokine administration, and use of memory T cells promise to overcome many of the limitations and pitfalls initially encountered with anti-CD20 mAb [3].

In this study, modified T cells were investigated to express an engineered anti-CD20scFvFc/CD28/CD3ζ receptor lysed CD20 positive Raji cells with higher efficiency, and it was capable to produce superior amounts of IFN-gamma and IL-2 compared to anti-CD20scFvFc transduced T cells. IFN-gamma produced by cytotoxic T lymphocyte is a critical cytokine for exerting antiviral, antimicrobial effect, and immune surveillance of tumors, which could directly inhibit proliferation and induce apoptosis of some malignancies in vivo and vitro through elusive mechanisms [13]. IL-2 is pivotal for survival of antigen-selected cytotoxic T cells via the activation of the expression of specific genes and development of T cell immunologic memory. Moreover, IL-2 has been shown to work in synergy with production of immunoglobulins and induce the proliferation and differentiation of natural killer cells [14]. It has been published that secretion of IFN-gamma and IL-2 plays an important role for a long lasting anti-tumor response of modified T cells [15]. Hence, superior secretion of IFN-gamma and IL-2 by anti-CD20scFvFc/CD28/CD3ζ recombinant gene modified T cells compared to anti-CD20scFvFc transduced T cells may achieve the dual benefit of enhanced ADCC and adaptive immune system engagement.

The B-cell restricted cell surface phosphor-protein CD20 is involved in many cellular signaling events including proliferation, differentiation, and apoptosis. So Rituximab can trigger and modify various intracellular signaling pathways in non-Hodgkin lymphoma B-cell lines, resulting in induction of apoptosis and chemosensitization. It is reported that the Fas-induced apoptotic pathway is involved in Rituximab mediated signaling transduction. This pathway activated by Fas is referred to as two type pathways. In type I pathway, initiator Caspases cleave and activate executor Caspases-3 directly. In type II pathway, also called mitochondrial pathway, is controlled by Bcl-2 family. The two pathways converge at the end by activating executor Caspases-3. Bcl-2 can inhibit apoptosis by preventing disruption of the mitochondria and the subsequent release of Cytochrome c. Consequently, overexpression of Bcl-2 has a protective effect against Fas-induced apoptosis in malignancies. It indicates potential therapeutic strategy for lymphoma treatment. Also, preclinical data from lymphoma cell lines and primary tumor samples indicate high efficacy of Bcl-2 inhibitor ABT-737 against lymphoma [16]. Caspase-3, a member of the Caspase family, has been found to integrate upstream signals into final execution of apoptosis. Its activity is an important predictor of apoptosis. Studies have shown unanimous results and clear evidence for this relationship. As expected, Rituximab-mediated apoptosis is thought to be a consequence of Caspase-3 activation, and data from patients with CLL also support this concept [17]. In this study, we observed that anti-CD20scFvFc/CD28/CD3ζ receptor grafted T cells could result in greater up-regulation of Fas expression, down-regulation of Bcl-2 and Caspase-3 activation in Raji cells compared to anti-CD20scFvFc receptor grafted T cells. From the secretion of cytokine and expression of apoptosis-related proteins in target cells, it manifested CD3ζ and CD28 co-stimulation signaling could synergistically enhance the target cytotoxicity and induction of apoptosis by gene modified T cells. Therefore this is expected to enhance the efficacy of the recombinant receptor approach, which can be used in the cellular immunotherapy of malignant diseases. Although we suppose it may overcome some limitations of anti-CD20 monoclonal antibody treatment from the promising results in present study, we anticipate the refinements in substantial research to validate its potential value in future.

## Conclusion

Our findings suggest that in addition to secretion of IFN-gamma and IL-2 according to the specific cytotoxicity against CD20 positive tumor cells by anti-CD20scFvFc/CD28/ζ receptor grafted T cells, Fas/FasL apoptotic pathway also contributes to anti-CD20scFvFc/CD28/ζ gene modified adoptive T cells-mediated cytotoxicity in vivo.

## Abbreviations

cTCRs: chimeric T cell receptors; scFv: single-chain variable fragment; ADCC: antibody-dependent cell-mediated cytotoxicity; CDC: complement-dependent cytotoxicity.

## Competing interests

The authors declare that they have no competing interests.

## Authors' contributions

YT, YZ and KY were equally involved in the design of the study and drafted the manuscript. YT, YZ and JD carried out most of the experiments. LJ, SZ, YH and PY participated in data organization and manuscript drafting. All authors read and approved the final manuscript.

## References

[B1] Prichard M Harris T Williams ME Densmore JJ Treatment strategies for relapsed and refractory aggressive non-Hodgkin's lymphoma Expert Opin Pharmacother 2009 10 6 983 995 10.1517/14656560902895715 19364248

[B2] Eshhar Z Waks T Gross G Schindler DG Specific activation and targeting of cytotoxic lymphocytes through chimeric single chains consisting of antibody-binding domains and the gamma or zeta subunits of the immunoglobulin and T-cell receptors Proc Natl Acad Sci USA 1993 90 2 720 724 10.1073/pnas.90.2.720 8421711 PMC45737

[B3] Till BG Press OW Treatment of lymphoma with adoptively transferred T cells Expert Opin Biol Ther 2009 9 11 1407 1425 10.1517/14712590903260785 19723016 PMC2776697

[B4] Stopeck AT Gessner A Miller TP Hersh EM Johnson CS Cui H Frutiger Y Grogan TM Loss of B7.2 (CD86) and intracellular adhesion molecule 1 (CD54) expression is associated with decreased tumor-infiltrating T lymphocytes in diffuse B-cell large-cell lymphoma Clin Cancer Res 2000 6 10 3904 3909 11051236

[B5] Krause A Guo HF Latouche JB Tan C Cheung NK Sadelain M Antigen-dependent CD28 signaling selectively enhances survival and proliferation in genetically modified activated human primary T lymphocytes J Exp Med 1998 188 4 619 626 10.1084/jem.188.4.619 9705944 PMC2213361

[B6] Yu K Hu Y Tan Y Shen Z Jiang S Qian H Liang B Shan D Immunotherapy of lymphomas with T cells modified by anti-CD20 scFv/CD28/CD3zeta recombinant gene Leuk Lymphoma 2008 49 7 1368 1373 10.1080/10428190802064958 18452062

[B7] Van Meerten T Hagenbeek A CD20-targeted therapy: a breakthrough in the treatment of non-Hodgkin's lymphoma Neth J Med 2009 67 7 251 259 19687518

[B8] Smith MR Rituximab (monoclonal anti-CD20 antibody): mechanisms of action and resistance Oncogene 2003 22 47 7359 7368 10.1038/sj.onc.1206939 14576843

[B9] Lenz G Wright G Dave SS Xiao W Powell J Zhao H Xu W Tan B Goldschmidt N Iqbal J Stromal gene signatures in large-B-cell lymphomas N Engl J Med 2008 359 22 2313 2323 10.1056/NEJMoa0802885 19038878 PMC9103713

[B10] Mounier N Briere J Gisselbrecht C Emile JF Lederlin P Sebban C Berger F Bosly A Morel P Tilly H Rituximab plus CHOP (R-CHOP) overcomes bcl-2--associated resistance to chemotherapy in elderly patients with diffuse large B-cell lymphoma (DLBCL) Blood 2003 101 11 4279 4284 10.1182/blood-2002-11-3442 12576316

[B11] Cooper LJ Ausubel L Gutierrez M Stephan S Shakeley R Olivares S Serrano LM Burton L Jensen MC Forman SJ DiGiusto DL Manufacturing of gene-modified cytotoxic T lymphocytes for autologous cellular therapy for lymphoma Cytotherapy 2006 8 105 117 10.1080/14653240600620176 16698684

[B12] Rosenblatt J Wu Z Vasir B Zarwan C Stone R Mills H Friedman T Konstantinopoulos PA Spentzos D Ghebremichael M Generation of tumor-specific T lymphocytes using dendritic cell/tumor fusions and anti-CD3/CD28 J Immunother 2010 33 155 166 10.1097/CJI.0b013e3181bed253 20145548 PMC2938173

[B13] Wall L Burke F Barton C Smyth J Balkwill F IFN-gamma induces apoptosis in ovarian cancer cells in vivo and in vitro Clin Cancer Res 2003 9 7 2487 2496 12855622

[B14] Handa K Suzuki R Matsui H Shimizu Y Kumagai K Natural killer (NK) cells as a responder to interleukin 2 (IL 2). II. IL 2-induced interferon gamma production J Immunol 1983 130 2 988 992 6294182

[B15] Maraskovsky E Chen WF Shortman K IL-2 and IFN-gamma are two necessary lymphokines in the development of cytolytic T cells J Immunol 1989 143 4 1210 1214 2501391

[B16] Oltersdorf T Elmore SW Shoemaker AR Armstrong RC Augeri DJ Belli BA Bruncko M Deckwerth TL Dinges J Hajduk PJ An inhibitor of Bcl-2 family proteins induces regression of solid tumours Nature 2005 435 7042 677 681 10.1038/nature03579 15902208

[B17] Byrd JC Kitada S Flinn IW Aron JL Pearson M Lucas D Reed JC The mechanism of tumor cell clearance by rituximab in vivo in patients with B-cell chronic lymphocytic leukemia: evidence of caspase activation and apoptosis induction Blood 2002 99 3 1038 1043 10.1182/blood.V99.3.1038 11807010

